# Chemical Composition and Digestibility of Major Feed Resources in Tanqua-Abergelle District of Central Tigray, Northern Ethiopia

**DOI:** 10.1155/2021/5234831

**Published:** 2021-06-15

**Authors:** Tikabo Gebremariam, Shumuye Belay

**Affiliations:** ^1^Department of Animal, Rangelands and Wildlife Sciences, Mekelle University, P.O. Box 231, Mekelle, Ethiopia; ^2^Mekelle Agricultural Research Center, Tigray Agricultural Research Institute, P.O. Box 492, Mekelle, Ethiopia

## Abstract

**Background:**

A detailed study on the feed quantity and quality is required to document the available feeds and their nutritional values.

**Aim:**

The study was aimed to investigate and document the chemical composition and *in vitro* dry matter digestibility of major feed resources available in Tanqua-Abergelle district of central Tigray, northern Ethiopia.

**Methods:**

Ten different feed resources were evaluated for their nutritive values. Representative feed samples were collected and prepared following appropriate procedures. The samples were subjected to analysis of chemical composition and *in vitro* dry matter digestibility (IVDMD) using proper scientific procedures.

**Results:**

Wide variations were observed in nutritive values of the investigated feeds. The highest crude protein (CP) was measured in *Atella* (15.90%) followed by green grass (13.20%), mill waste (10.90%), groundnut straw (9.18%), and cowpea straw (8.11%) in descending order. Mill waste (11.84 MJ/kg DM) and *Atella* (11.81 MJ/kg DM) had the highest metabolizable energy (ME) followed by green grass (9.83 MJ/kg DM), groundnut straw (9.28 MJ/kg DM), *Teff* straw (8.56 MJ/kg DM), and cowpea straw (8.39 MJ/kg DM) in that order. The highest NDF was recorded in groundnut null (79.80%) and the lowest NDF in mill waste (35.00%) and *Atella* (40.60%). The highest IVDMD was seen in mill waste (81.43%) and *Atella* (81.21%) and the lowest in groundnut hull (39.95%).

**Conclusion:**

The nonconventional feeds have moderate protein and reduced fiber contents, and thus, they can be utilized as supplement for poor-quality feeds. These feeds need further investigation using animals to substantiate the current study.

## 1. Introduction

Food and nutrition security is the top priority of the developing countries. Livestock play multiple roles in economic growth, food security, and poverty reduction. Among the African nations, Ethiopia is well known for possession of huge livestock resources. The country is a habitat for about 60.39 million cattle, 31.30 million sheep, 32.74 million goats, and 56.06 million poultry [1]. Large numbers of animals are kept under the dominant mixed crop-livestock farming system. Livestock is central for the livelihood of many Ethiopian smallholder farmers that majorly live in rural areas. Livestock production offers many economic and social functions to the rural people as a source of varieties of foods, income, saving, drought power, and compensation [2]. At the national level, livestock contribute about 15–17% of the national gross domestic product (GDP), 35–40% of the agricultural GDP, and 37–87% of the household incomes [3].

Despite its magnificent importance, the livestock sector is challenged by many complicated technical and nontechnical constraints. Feed shortage is the key problem in livestock production, particularly in the case of smallholder farmers. The livestock production is operated under feed deficit condition. There is mismatch between livestock feed demand and supply. Empirical evidences show that the feed deficit amounts about 36–50% in dry matter, 18% energy, and 34–38% protein [4, 5]. Similarly, a study reported negative livestock feed balance in Ethiopia with a deficit of 9% DM, 45% energy, and 42% protein [6]. This implies that the feed improvement levies extraordinary attention from the government and concerned bodies. Of course, for the last 25 years, tremendous efforts have been made in feed development in the country; however, it was accompanied with less success story, leaving unsolved the feed shortage problem faced by livestock producers. The feed deficit problem is getting severe with time as the livestock number is increasing along with human population. This can disrupt the normal functions of the livestock production system and bring about ecological imbalance due to uncontrolled and excessive use of communal grazing lands [7]. This entails devising and implementing strategic intervention options that can achieve feed supply improvement to maintain feed balance.

Crop residues, natural pastures, crop stubble, and hay are the major livestock feed resources in the Tigray Region, northern Ethiopia [8–10]. The availability and distribution of these feed resources differ with the season, location, and farming system. Likewise, apparent variations are seen in the nutritional quality of these feed resources. These variations of nutritional contents could be attributed to species, varieties, age of the plant, soil type, agronomic practices, climatic conditions, and other environmental factors [11, 12]. A detailed study on the feed quantity and quality is required to document the available feeds and their nutritional values. In this regard, an immense gap is seen in the nutritional profile of local feed resources. There is also wide recognized variation on the available nutrition data and information for these feeds. Nutritional profiles are necessitated for efficient utilization of locally available feed resources in a given area. Hence, this study was carried out with the aim to investigate and document the chemical composition and *in vitro* dry matter digestibility of major feed resources available in Tanqua-Abergelle district of central Tigray, northern Ethiopia.

## 2. Materials and Methods

### 2.1. Descriptions of the Study Area

The study was undertaken in Tanqua-Abergelle district of central Tigray, northern Ethiopia. The study area is located at 13° 14′ 06″ N latitude and 38° 58′ 50″ *E* longitude. The altitude varies from 900 to 1800 m above sea level with an annual rainfall of 370–700 mm. The average minimum and maximum temperatures are 21°C and 41°C, respectively. The area is classified as hot to warm submoist with 95% lowland and 5% midaltitude. The soil is described dominantly as vertisol with 8.33% clay, 16.33% sandy, 22.14% loam, 47.40% silt, and 5.8% silt-loam textures [13]. Smallholder farmers of the study area are engaged in rearing different livestock types in the mixed crop-livestock farming system. Cattle, sheep, goats, and donkeys are dominantly raised by the smallholder farmers. Small ruminant production is the predominant system in the area with considerable economic and social functions. The livestock serve as sources of power, food, income, manure, and savings. *Sorghum*, maize, *Teff* (*Eragrostis tef*), finger miller, pulses, and groundnut are the dominant crop types cultivated in the area [13]. Crop residues, natural pastures, crop stubble, and hay are the major feed resources fed to livestock [9].

### 2.2. Sample Feed Collection and Preparation

**Figure 1:**
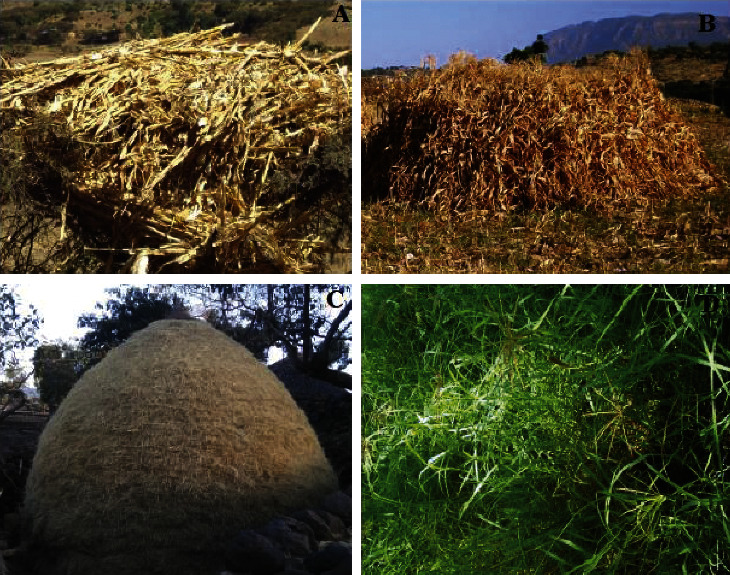
Some of the feeds available in the study area: (a) *Sorghum* stover, (b) maize stover, (c) Teff straw, and (d) green grass.

Feed samples were collected from the representative sites of the study district (Figure 1). Ten different feed samples were collected from major feed resources based on their importance and contribution as reported in the same study [9]. Selection of feed types was carried out based on the information obtained from household interviews and focus group discussions. The feeds were collected from different parts of the study areas following appropriate procedures. The feed samples were labeled carefully at the field to have full information. Three samples were taken from each feed type for laboratory analysis. Partial dry matter analysis was conducted for these feed samples such as *Atella* with high moisture contents using the air-drying method. The samples were dried at 65°C for 72 hours, and they were ground in a Willy mill to pass through a 1 mm sieve. The milled samples were reserved in a proper place at Mekelle University for a limited time pending for chemical analysis. Thereafter, the samples were sent to the International Livestock Research Institute (ILRI), Addis Ababa, Ethiopia, for laboratory analysis.

### 2.3. Chemical Analysis of Feeds

The feed samples were subjected to chemical analysis for major nutrients. Dry matter (DM), crude protein (CP), and ash contents were analyzed by the standard methods in [14]. The neutral detergent fiber (NDF), acid detergent fiber (ADF), and acid detergent lignin (ADL) were determined based on the method of [15]. Hemicellulose and cellulose contents were calculated by differences as NDF−ADF and ADF−ADL, respectively. Similarly, the organic matter (100−ash), soluble matter (100−NDF), and soluble carbohydrates (100−NDF−CP−ash) were computed using formulas. *In vitro* dry matter digestibility (IVDMD) was determined by the Tilley and Terry method as modified in [16]. Common macro- and trace elements such as Ca, Fe, Mn, Cu, and Zn were determined using atomic absorption and mass spectrometry procedures in [14]. Metabolizable energy (ME) was computed following the formula recommended in [17] as ME (MJ/kg DM) = 0.17∗IVDMD (%) −2.0. All analyses were made in triplicates.

### 2.4. Data Statistical Analysis

The data on chemical composition and *in vitro* dry matter digestibility (IVDMD) were subjected to the one-way analysis of variance (ANOVA) using General Linear Model (GLM) procedures in [18]. A statistical test (*p* < 0.05) was used to compare the individual means. Tables were used to organize and summarize results.

## 3. Results

### 3.1. Chemical Composition of Major Feed Resources

The investigated major feed resources include crop residues, natural mixed hay, green grass, and nonconventional feeds. A total of ten feed samples were evaluated for their nutritive values in the present study.  Table 1 provides information about the chemical composition of the feeds. As shown in the table, the dry matter (DM) of straws varied from 91.37% to 94.61%, stovers 94.61 to 96.29%, and hay 96.23%. Groundnut hull and mill waste had 90.90 and 89.90% DM, respectively. The lowest DM was recorded for *Atella* (13.23%) followed by green grass (30.49%). The highest crude protein (CP) was measured in *Atella* (15.90%) followed by green grass (13.20%), mill waste (10.90%), groundnut straw (9.18%), and cowpea straw (8.11%) in descending order. Hay (6.29%) had higher CP than *Teff* straw (4.43%) and stovers (2.76-4.91%). The highest ash content was found in green grass (12.30%) and the lowest in mill waste (3.80%) while others were in between. *Atella* is the residue of local brewery.

The soluble matter, soluble carbohydrates, and metabolizable energy contents of the major feed resources are presented in Table 2. The soluble carbohydrates content was recorded as the highest in mill waste (50.30%) followed by groundnut straw (38.68%) and *Atella* (37.50%). The lowest soluble carbohydrates were found in green grass (6.60%). Mill waste (11.84 MJ/kg DM) and *Atella* (11.81 MJ/kg DM) had the highest metabolizable energy (ME) followed by green grass (9.83 MJ/kg DM), groundnut straw (9.28 MJ/kg DM), *Teff* straw (8.56 MJ/kg DM), and cowpea straw (8.39 MJ/kg DM) in that order. The lowest ME content was observed in stovers (7.22-7.68 MJ/kg DM), hay (6.70 MJ/kg DM), and groundnut hull (4.79 MJ/kg DM) in reducing order.

### 3.2. Fiber Fractions and Digestibility of Feeds

The fiber fractions and *in vitro* dry matter digestibility (IVDMD) of the major feed resources are presented in Table 3. A wide variation was observed in the cell wall contents of the feeds. The highest NDF was measured in groundnut null (79.80%) followed by stovers (71.60-76.15%), *Teff* straw (75.05%), hay (73.72%), cowpea straw (62.69%), and groundnut straw (46.67%) in that order. The lowest NDF was seen in mill waste (35.00%) and *Atella* (40.60%). The highest ADF was observed in groundnut hull (74.30%) and the lowest in mill waste (13.40%) while the others are in between. The ADL content varied from 31.80% (groundnut hull) to 3.30% (mill waste). In the case of *in vitro* dry matter digestibility (IVDMD), the highest value was found in mill waste (81.43%) and *Atella* (81.21%) followed by green grass (69.58%), groundnut straw (66.33%), *Teff* straw (62.10%), and cowpea straw (61.09%) in reducing order. The lowest IVDMD value was observed in stovers (54.21-56.97%), hay (51.15%), and groundnut hull (39.95%) in that order.

### 3.3. Mineral Profile of Feeds

The macro- and micromineral contents of major feed resources are given in Table 4. As shown in the table, there is a wide variation in mineral contents of the different feed resources. The calcium content varied from 15.14 g/kg DM (groundnut straw) to 0.12 g/kg DM (mill waste). The highest iron content was observed in *Atella* (3.37 g/kg DM) while the lowest was in cowpea straw (0.11 g/kg DM). *Atella* (0.19 g/kg DM) had the highest zinc content while groundnut hull (0.07 g/kg DM) had the lowest value. The content of copper varied from 9.59 mg/kg DM (groundnut straw) to 4.55 mg/kg DM (*Atella*). *Teff* straw (78.41 mg/kg DM) had the highest manganese content and the lowest value belonged to *Atella* (0.06 mg/kg DM). *Atella* contained high iron and zinc minerals but low copper and manganese. Groundnut straw was rich in both calcium and copper minerals. *Teff* straw was blessed with high manganese when compared with other feeds. *Teff* straw was also rich in iron content following *Atella*.

## 4. Discussion

### 4.1. Chemical Composition

The study showed that there is considerable variation in the chemical composition of the available feeds. The variation could be attributed to the plant species, plant variety, soil type, plant fraction, plant management, and other factors [11, 12]. Crude protein content was higher in *Atella* (15.90%), green grass (13.20%), and mill waste (10.90%) as compared to the other feed resources. According to [19], feeds that have <12%, 12–20%, and >20% CP are classified as low, medium, and high protein sources, respectively. Based on this classification, *Atella* is regarded as medium protein source, and it does have the potential to serve as protein supplement to the poor-quality tropical feeds. *Atella* is a by-product obtained from the production of tella (local brewery). The production process of tella includes fermentation of cereal crops, mostly sorghum, maize, and finger millet, with *Saccharomyces cerevisiae*, and malt and gesho (*Rhamnus prinoides*) are added as substitutes for hops [20]. *Atella* is widely used as supplement for dairy cows in combination with niger cake and wheat bran [21, 22]. Milk yield increment (14%) was reported when barley-straw-based dairy cows were supplemented with *Atella* in association with niger cake and wheat bran [22]. The current CP value of *Atella* was lower than 21.80% [23] and 20% [24]. Scientific evidence shows that above 7% CP is required for microbial protein synthesis in the rumen that can support at least the maintenance requirement of ruminants [12, 25, 26]. Straw/stover of cereal crops are below this critical crude protein level, and they must be supplemented with protein sources.

Straws of pulse and oil seed crops have relatively better protein concentration when compared to hay, cereal straws, and stovers. The OM (90.90%) contents of groundnut straw were lower when compared with the finding in [27] with 94.67% OM. The CP value (9.18%) of groundnut straw was higher than 8.08% reported in [27] but lower than 11.1% in [28]. The ash value (5.47%) of groundnut straw found in the present study was comparable with the result in [27], 5.33%, but much lower than that in [28], 16.3%. The DM of groundnut hull (90.90%) was near to the finding in [29] with 91.6% DM for the same feed. The CP of groundnut hull (7.10%) was similar with that in [29], 7.0%. The same authors reported higher ash content (5.2%) than the current study (2.70%) for groundnut hull.

The content of soluble carbohydrates was higher in groundnut straw and *Atella*, indicating their potential use as source of energy for tropical animals based on poor-quality feeds. On the contrary, the lowest soluble carbohydrate content of green grass shows its low energy source in the animal diet. Green grasses are noted for their good protein, mineral, and vitamin sources in the animal feeding system [12]. A study argues that the feeding potential of roughage feeds is significantly affected by the proportion of soluble and insoluble fraction that affects fermentation rate of feeds in rumen [30]. Likewise, metabolizable energy (ME) was higher in mill waste and *Atella*, indicating their potential source of energy to poor-quality feeds. These nonconventional feeds have above minimum energy requirement for meat and dairy animals. Natural mixed hay, straws/stovers of different crops, and groundnut hull contain low energy below the critical level. The lowest energy density at which sheep does not lose weight is between 8 and 10 MJ ME/kg DM [25]. The current ME of *Atella* (11.81 MJ/kg DM) was higher when compared with 10.00 MJ ME/kg DM (2.39 Mecal ME/kg DM) reported in [23] but lower than 18.20 MJ ME/kg DM (22.2 MJ GE/kg DM) in [21] and 16.29 MJ ME/kg DM (4.75 kcal GE/kg DM) in [31]. The variation in energy values of the different research could be attributed to the crop species/variety used, fermentation process, climatic conditions, and other factors. *Atella* was reported to have 19.9 MJ/kg DM gross energy in a review result in [24]. The ash content of *Atella* (6.00%) was comparable with the findings in [23] (5.8%) and [24] (5.4%). The ME content of groundnut straw (9.28 MJ/kg DM) was comparable with the finding of 9.4 MJ/kg DM in [28]. The ME content of groundnut hull (4.79 MJ/kg DM) was higher when compared with the 2.7 MJ/kg DM reviewed in [29].

### 4.2. Fiber Fraction and Digestibility

Cell wall fractions (NDF and ADF) were higher in crop residues, hay, and groundnut hull when compared to the other feeds. The fiber content of *Teff* straw was comparable with that in [32] (77.65%) and [33] (77.5%). Similarly, the NDF value of hay (73.72%) was comparable with the reports in [34] with 72.20%, but lower than that in [35] with 79.40%. The proportion of fiber content in hay is greatly influenced by the stage of maturity at cutting and postharvest management [12]. High fiber content (above 55% NDF) limits the feed intake of animals by filling the gut [26]. Hence, the high fiber content can hamper the feed intake of animals, resulting in reduced animal performance. According to [36], the maximum NDF content that can negatively affect feed consumption is estimated to be as high as 70–75% for mature beef cows and as low as 15–20% for finishing ruminant animals. Based on this fact, feeds having above 70% fiber content could have a negative impact on feed utilization and animal performance. On the contrary, feeds with less cell wall components such as mill waste and *Atella* encourage consumption of feeds by animals. It is indicated that fiber fractions (NDF and ADF) are the key responsible for limited forage intake and digestibility due to rumen fill and strong relationship with rumination [37]. The NDF and ADF values found in *Atella* in the present study were lower as compared with 54.0% NDF and 29.0% reported in [23]. The NDF (46.67%), ADF (36.11%), and ADL (7.55%) values of groundnut straw obtained in the current study were lower when compared with the findings in [27] with 70.70% NDF, 64.33% ADF, and 11.62% ADL but higher than that in [28] with 35.2% NDF and 29.1% ADF. The fiber content of groundnut hull in the present study was higher than the findings in [29] with 66.4% NDF and 56.4% ADL.

A wide variation (35–81%) was seen in the *in vitro* dry matter digestibility (IVDMD) of the feeds investigated in the current study. The IVDMD was found to be higher in most investigated feeds except hay, stovers, and groundnut hull. Mill waste and *Atella* had higher DM digestibility when compared to green grass, oil crop straw, pulse straw, cereal straw, and stover. The high digestibility values of *Atella* and mill waste are attributed to the high protein concentration as well as to the low fiber contents. It is well established fact that digestibility is positively correlated with nitrogen consumption and negatively correlated with fiber content [12, 26]. Feeds with high digestibility coefficient secure better utilization by animals and vice versa. The organic matter digestibility of *Atella* was reported to be 54.8% in [24]. The same report also found 66.1% nitrogen digestibility for *Atella* in ruminant animals. In [23], 60.4% dry matter digestibility was found for *Atella* while it was 81.21% in this study. The lowest digestibility seen in groundnut hull can be explained by the high fiber fractions. The DM digestibility of groundnut straw (66.33%) was lower when compared with the review result of 73.1% OM digestibility reported in [28] for groundnut straw. A lower OM digestibility was reported by [29] for groundnut hull (20.1%) when compared with the current study with 39.95% DM digestibility.

### 4.3. Mineral Profiles

The feeds available in the study area varied in their mineral contents. All the investigated feeds, except groundnut straw, failed to fulfill the minimum critical level of calcium as the major mineral for optimum rumen function based on the recommendation in [12]. Calcium is the most abundant mineral element in the animal body as the main constituent of the skeleton and teeth. Among the feeds, groundnut straw (15.14 g/kg DM) had the highest calcium content which is optimum for normal rumen function and feed consumption (15 g/kg DM). Maintaining the optimum ratio of calcium and phosphorous is quite important for normal body function. High calcium content in the diet could interfere with the absorption and metabolism of phosphorous in the animal body, if the accepted safe range ratio of calcium to phosphorous (1 : 1 to 2 : 1) is not carefully manipulated in the diet [12]. On the other hand, feeds such as mill waste had low calcium value which is below the critical level (15 g/kg DM). Deficiency of calcium can cause bone deformation in growing animals, milk fever in milking cows, and abnormal body function [12]. These feeds need supplementation with calcium sources such as bone meal, green leafy crops, ground limestone, and mineral mix. The calcium content of *Atella* (0.82 g/kg DM) was lower than that in [24] where 6.2 and 5.4 g/kg DM calcium and phosphorous, respectively, were found in the review results. Among the examined feeds, groundnut straw had more calcium and copper minerals. The calcium content of groundnut straw (15.13 g/kg DM) was almost near to the review results in [28] with 14.6 g/kg DM calcium and 2.0 g/kg phosphorus. In [29], groundnut hull was found to have 2.4 g/kg DM calcium, 0.7 g/kg DM phosphorous, 38 mg/kg DM manganese, 64 mg/kg DM zinc, 15 mg/kg DM copper, and 210 mg/kg DM iron.

Trace minerals play an important role in the body function of farm animals. They are required in a small amount in the animal body or in the diet. In the current study, most of the feeds satisfy the minimum requirement of trace elements such as iron, zinc, copper, and manganese. All the feeds (124–242 mg/kg DM) owned above optimum requirement of iron (20–80 mg/kg DM), while *Atella* (3372 mg/kg DM) and *Teff* straw (1009 mg/kg DM) had extreme high iron values. In [12], it was noted that iron toxicity is not a common problem in farm animals. The zinc content of most feeds (12.19–44.87 mg/kg DM) was within the range of the recommended body requirement (10–50 mg/kg DM) while *Atella* (190 mg/kg DM) showed excess and groundnut hull (7.75 mg/kg DM) below the critical level. Farm animals are believed to possess a high tolerance to excess zinc in their diet. All the current feeds (5.56-9.59 mg/kg DM) contained above the optimum range (1–5 mg/kg DM) of copper requirement. *Atella* (4.55 mg/kg DM) and mill waste (4.65 mg/kg DM) had copper content within the optimum demand for ruminant animals. Scientific evidence shows that excess ingestion of copper salt by animals causes toxicity due to accumulation in the liver and, thus, considerable care is required in this regard [12]. All the feeds (17.33–78.41 mg/kg DM) except *Atella* (0.06 mg/kg DM) had excess manganese while the optimum amount required in the diet is 0.2–0.5 mg/kg DM.

## 5. Conclusions

The current study gave information on nutritional profiles of major feed resources available in Tanqua-Abergelle district of central Tigray. A wide variation is recognized in the nutritive values of the feeds. Crop residues and hay have poor feeding values, demanding supplementation with good quality feeds. Nonconventional feeds such as *Atella* and mill waste have moderate protein and reduced fiber contents, and they can serve as potential protein sources to improve the nutritive value of poor-quality tropical feeds in the animal feeding system. These feeds need further *in vivo* investigation using animals to substantiate the present study.

## Figures and Tables

**Table 1:** Chemical composition of major feed resources available in the study area.

Feed type	DM (%)	OM (% DM)	CP (% DM)	Ash (% DM)
*Teff* straw	91.37^ab^	92.85	4.43^d^	7.15^b^
*Sorghum* stover	94.84^a^	95.85	2.76^e^	4.15^c^
Maize stover	96.29^a^	95.62	4.91^d^	4.38^c^
Cow pea straw	94.61^a^	96.62	8.11^bc^	3.38^cd^
Groundnut straw	91.89^ab^	94.53	9.18^bc^	5.47^c^
Groundnut hull	90.90^b^	97.30	7.10^c^	2.70^d^
Natural mixed hay	96.23^a^	92.32	6.29^c^	7.68^b^
Green grass	30.49^c^	87.70	13.20^ab^	12.30^a^
*Atella* ^*∗*^	13.23^d^	94.00	15.90^a^	6.00^b^
Mill waste	89.80^b^	96.20	10.90^b^	3.80^cd^
SEM	9.63	0.89	1.29	0.89
*P* value	0.000	0.056	0.022	0.044

^*∗*^Residues of local brewery; DM = dry matter; OM = organic matter; CP = crude protein; SEM = standard error of mean.

**Table 2:** Soluble carbohydrates and energy contents of major feed resources (% of DM).

Feed type	Soluble matter	Soluble carbohydrates	ME (MJ/kg DM)
*Teff* straw	24.95^d^	13.37^d^	8.56^b^
*Sorghum* stover	28.40^cd^	21.49^cd^	7.68^c^
Maize stover	23.85^d^	14.56^d^	7.22^c^
Cowpea straw	37.31^c^	25.82^c^	8.39^b^
Groundnut straw	53.33^b^	38.68^b^	9.28^ab^
Groundnut hull	20.20^e^	10.40^de^	4.79^d^
Natural mixed hay	26.28^d^	12.31^d^	6.70^c^
Green grass	32.10^cd^	6.60^e^	9.83^ab^
*Atella* ^*∗*^	59.40^ab^	37.50^b^	11.81^a^
Mill waste	65.00^a^	50.30^a^	11.84^a^
SEM	5.12	4.61	0.69
*P* value	0.000	0.000	0.012

^*∗*^Residues of local brewery; DM = dry matter; ME = metabolizable energy; MJ = megajoule; SEM = standard error of mean.

**Table 3:** Fiber fractions and digestibility of major feed resources (% of DM).

Feed type	NDF	ADF	ADL	Cellulose	Hemicellulose	IVDMD
*Teff* straw	75.05^ab^	48.74^b^	6.58^bc^	35.01^ab^	26.31^b^	62.10^bc^
*Sorghum* stover	71.60^b^	45.75^bc^	5.98^c^	35.62^ab^	25.85^b^	56.97^c^
Maize stover	76.15^ab^	47.53^b^	5.23^c^	37.92^a^	28.62^a^	54.21^c^
Cowpea straw	62.69^c^	45.77^bc^	8.94^b^	33.45^ab^	16.92^cd^	61.09^bc^
Groundnut straw	46.67^d^	36.11^c^	7.55^b^	23.09^c^	10.56^d^	66.33^b^
Groundnut hull	79.80^a^	74.30^a^	31.80^a^	39.80^a^	5.50^e^	39.95^e^
Natural mixed hay	73.72^b^	44.10^bc^	6.08^bc^	30.34^b^	29.62^a^	51.15^d^
Green grass	67.90^bc^	35.90^c^	6.00^bc^	17.60^d^	32.00^a^	69.58^b^
*Atella* ^*∗*^	40.60^de^	19.30^d^	8.40^b^	4.90^e^	21.30^c^	81.21^a^
Mill waste	35.00^e^	13.40^e^	3.30^d^	6.30^e^	21.60^c^	81.43^a^
SEM	5.12	5.32	2.58	4.07	2.71	4.09
*P* value	0.004	0.003	0.021	0.006	0.018	0.006

^*∗*^Residues of local brewery; DM = dry matter; NDF = neutral detergent fiber; ADF = acid detergent fiber; ADL = acid detergent lignin; IVDMD = *in vitro* dry matter digestibility; SEM = standard error of mean.

**Table 4:** Macro- and micromineral profile of major feed resources.

Feed type	Calcium (g/kg DM)	Manganese (gm/kg DM)	Iron (gm/kg DM)	Zinc (gm/kg DM)	Copper (gm/kg DM)
*Teff* straw	4.54^bc^	78.41^a^	1009.25^b^	28.61^c^	8.56^a^
*Sorghum* stover	3.29^c^	54.27^b^	181.71^cd^	13.23^d^	5.65^b^
Maize stover	1.16^d^	17.33^e^	116.62^de^	12.73^d^	6.00^b^
Cowpea straw	6.72^b^	47.56^c^	105.53^e^	16.03^d^	5.88^b^
Groundnut straw	15.14^a^	46.02^c^	213.89^c^	12.19^d^	9.59^a^
Groundnut hull	0.24^e^	36.37^d^	124.91^d^	7.75^e^	6.32^b^
Natural mixed hay	4.04^bc^	31.32^d^	133.87^d^	14.33^d^	8.65^a^
Green grass	3.13^c^	17.40^e^	242.94^c^	41.52^b^	6.26^b^
*Atella* ^*∗*^	0.82^e^	0.06^f^	3372.31^a^	190.63^a^	4.55^c^
Mill waste	0.12^e^	36.45^d^	143.49^d^	44.87^b^	4.65^c^
SEM	1.41	6.96	3.23	17.42	0.54
*P* value	0.026	0.000	0.007	0.000	0.016

^*∗*^Residues of local brewery; DM = dry matter; SEM = standard error of mean.

## Data Availability

Data can be made available upon request from the corresponding author.
